# Effect of Laminating Pressure on Polymeric Multilayer Nanofibrous Membranes for Liquid Filtration

**DOI:** 10.3390/nano8050272

**Published:** 2018-04-24

**Authors:** Fatma Yalcinkaya, Jakub Hruza

**Affiliations:** 1 Department of Nanotechnology and Informatics, Institute of Nanomaterials, Advanced Technologies and Innovation, Technical University of Liberec, Studentska 1402/2, 46117 Liberec, Czech Republic; jakub.hruza@tul.cz; 2 Institute for New Technologies and Applied Informatics, Faculty of Mechatronics, Studentska 1402/2, 46117 Liberec, Czech Republic

**Keywords:** nanofiber, lamination, water filtration

## Abstract

In the new century, electrospun nanofibrous webs are widely employed in various applications due to their specific surface area and porous structure with narrow pore size. The mechanical properties have a major influence on the applications of nanofiber webs. Lamination technology is an important method for improving the mechanical strength of nanofiber webs. In this study, the influence of laminating pressure on the properties of polyacrylonitrile (PAN) and polyvinylidene fluoride (PVDF) nanofibers/laminate was investigated. Heat-press lamination was carried out at three different pressures, and the surface morphologies of the multilayer nanofibrous membranes were observed under an optical microscope. In addition, air permeability, water filtration, and contact angle experiments were performed to examine the effect of laminating pressure on the breathability, water permeability and surface wettability of multilayer nanofibrous membranes. A bursting strength test was developed and applied to measure the maximum bursting pressure of the nanofibers from the laminated surface. A water filtration test was performed using a cross-flow unit. Based on the results of the tests, the optimum laminating pressure was determined for both PAN and PVDF multilayer nanofibrous membranes to prepare suitable microfilters for liquid filtration.

## 1. Introduction

Electrospun polymeric nanofiber web has gained increasing importance in the production of engineered surfaces with sub-micron to nano-scale fibers. The widely employed areas of electrospun nanofibers are tissue engineering [1,2], wound healing [3], drug delivery systems [4], composites [5], solar cells [6], protective clothing [7], lithium-ion batteries [8,9], sensors [10,11,12], gas sensors and separators [13,14], and air and water filtration [15,16,17,18,19], owing to their high surface-area-to-volume ratio, highly porous structure and extremely narrow pore size. The main factor influencing the application of nanofibers is their mechanical properties. An electrospun nanofiber has very poor mechanical strength due to low contact and adhesion between the fibers. 

Several methods have been developed to provide suitable mechanical strength to electrospun nanofibers. One of the most common methods is to blend several polymers, the advantage of which is that it is easy and low-cost. However, it is necessary to use polymers which can dissolve in the same solvent system, of which there are only a few [20,21]. In another method, lamination was achieved using an epoxy composite. In this method, an electrospun layer was laid on the epoxy/curing agent in a mold and then the curing process was performed for a period of 16 h [22]. Nanofiber reinforced nanomaterials such as carbon nanotubes and graphene represent another route for improving the mechanical strength of nanofibers. However, this method is costly and in some cases has a low efficiency [23,24]. Charles et al. [25] used a dip coating method to improve the mechanical strength of nanofibers. They described the mechanical properties of a composite system comprising hydroxyapatite (HA)-coated poly (l-lactic acid) (PLLA) fibers in a poly (ε-caprolactone) (PCL) matrix. A biomimetic method was used to coat the fibers with HA, and a dip-coating procedure served for the application of PCL to the coated fibers. The composite was formed into a bar using compression molding at low temperatures. The disadvantage of this method is that it is a time- and chemical-consuming procedure. Xu et al. [26] developed a self-reinforcing method to enhance the strength of polycarbonate (PC) membranes. In this method, the PC nanofibers were immersed into a solvent (30%) and non-solvent (70%) mixture, which resulted in enhancement in the strength (128%) owing to the fusion of junction points. The entire porous structure on the PC nanofibers was destroyed, which greatly impaired the application of the membranes. The thermal lamination method is one of the most reliable, repeatable, time-saving, environmentally friendly and cost-effective methods used to adhere two surfaces. In this method, an adhesive polymer or web is usually applied between two surfaces. Using heat and pressure, the surfaces adhere to each other. There is a large amount of research related to improving the strength of nanofibers; however, the number of reports is still limited compared to those dealing with lamination technology. Jiricek [27,28] and Yalcinkaya et al. [29,30] used a bi-component polyethylene (PE)/polypropylene (PP) spunbond as a supporting layer for nanofiber layers. A fusing machine was used for the lamination process. A nanofiber layer was adhered on the outer surface of the bi-component due to the low melting point of PE. The resultant multilayer nanofibrous membranes were used for micro and nanofilters. The supporting material and the density of the nanofibers have an influence on the water and air permeability of the multilayer materials. Yoon et al. [7] laminated nanofibers with various densities of polyurethane (PUR) fiber onto different textile surfaces using an adhesive web. The results showed that the various multilayer nanofibrous membrane structure designs had a considerable influence on the degrees of breathability and waterproofness of the textile surfaces. In our previous work [31], it was observed that both the supporting material and the density of the nanofiber web have an influence on the water permeability of the multilayer nanofibrous membranes. The lower area weight with open structure supporting materials has higher water flux and permeability. Kanafchian et al. [32] used a heat-press technique to laminate the polyacrylonitrile (PAN) nanofiber on a polypropylene spunbond at various laminating temperatures. It was observed that, when the applied temperature is lower than the melting point of polypropylene spunbond, the nanofiber web remains unchanged. Moreover, the increase in temperature increased the adhesion between nanofiber while decreasing the air permeability. 

Although the system and parameters of the electrospinning process have been well analyzed, there is still a lack of information about a proper lamination technique for nanofiber webs. So far, mainly the effect of temperature on the lamination of electrospun nanofiber webs has been investigated using heat-press methods [32,33,34]. Yao et al. [33] studied the effect of the heat-press temperature, pressure, and duration on the morphological and mechanical characteristics of an electrospun membrane for membrane distillation. The results showed that the temperature and duration of the heat-press play more important roles than the pressure in the heat-press treatment. However, the pressure varied between 0.7 and 9.8 kPa at 150 °C during a 2-h period, which is time- and energy-consuming and therefore a more comprehensive parametric study is required. The aim of this study is to consider the influence of laminating pressure on the properties of multilayer nanofibrous membranes. Polyvinylidene (PVDF) and polyacrylonitrile (PAN) are the most commonly used nanofiber layers owing to their chemical and thermal stability. Herein, both polymers were electrospun using a semi-industrial scale nanofiber production method. The nanofiber layers were laminated onto a nonwoven surface to improve their mechanical strength via the heat-press method, which is easy to scale and is an energy-saving method at various applied pressures. Other conditions, such as temperature and the duration of lamination, were kept stable. The effect of the laminating pressure on the nanofiber web has not yet been well reported. To investigate the effect of the pressure on the nanofiber/laminate process, surface morphology under an optical microscope, the minimum bursting pressure, air permeability, water permeability and contact angle tests were applied. Our objective was to optimize the lamination technology and produce multilayer nanofibrous membranes for suitable application in liquid filtration. Another novelty of this paper is that nanofiber layers were produced by using the industrial equipment, and that the layers strongly adhered on the supporting layer without any damage using various lamination pressures to improve their application in liquid filtration.

## 2. Materials and Methods

### 2.1. Preparation of Nanofibre Webs

**Figure 1:**
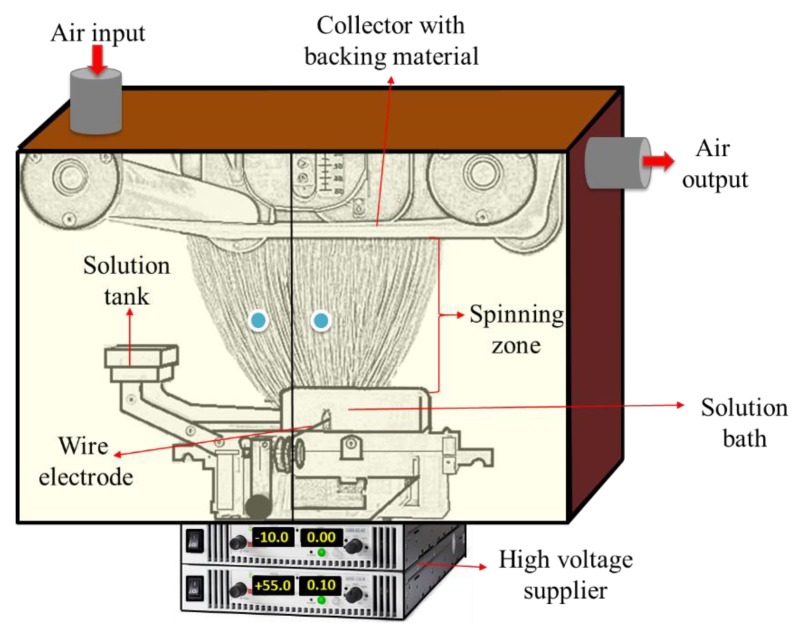
Schematic diagram of an electrospinning unit.

13 wt. % PVDF (Solef 1015, Bruxelles, Belgium) was prepared in *N*,*N*-dimethylacetamide (DMAC) and 8 wt. % PAN (150 kDa H-polymer, Elmarco, Liberec, Czech Republic) was prepared in *N*,*N*-dimethylformamide. The solvents were purchased from Penta s.r.o. (Prague, Czech Republic). The solutions were stirred overnight using a magnetic stirrer. Nanofiber webs were prepared using the semi-industrial Nanospider electrospinning device (Elmarco, Liberec, Czech Republic) as shown in Figure 1. 

The solution is placed in a solution tank, which is a closed system and connected to a solution bath. The wire electrode passes along a metal orifice in the middle of the solution bath. The solution bath is moved back and forward to feed the surface of the wire electrode. The solution bath feeds the polymer solution on a moving stainless steel wire. The speed of the bath is 240 mm/s. A high voltage supplier is connected to a positively charged wire electrode (55 kV). A second wire electrode, which is connected to a negatively charged voltage supplier (−15 kV), is placed on the top of the spinning unit. A conveyor backing material is placed between the two electrodes. The spinning takes place between the two electrodes. The nanofiber web is collected on baking paper moving in front of the collector electrode. The distance between the electrodes is 188 mm. The distance between the second electrode and the supporting backing material is 2 mm. The speed of the backing material for PAN and PVDF is 15 mm/min and 20 mm/min, respectively. The amount of solution on the wire is controlled with the speed of the solution bath, the diameter of the wire (0.2 mm) and the diameter of the metal orifice (0.6 mm) in the middle of the solution bath. No solution dipping was observed. An air conditioning unit is used to control the humidity and temperature of the spinning in a closed chamber. The temperature and humidity of the input air are set to 23 °C and 20% by the air-conditioning system. The volumes of air input and output are 100 and 115 m^3^/h, respectively. The area weight of the PVDF and PAN nanofibers was set at 3 g/m^2^.

### 2.2. Lamination of Nanofibre Webs

**Figure 2:**
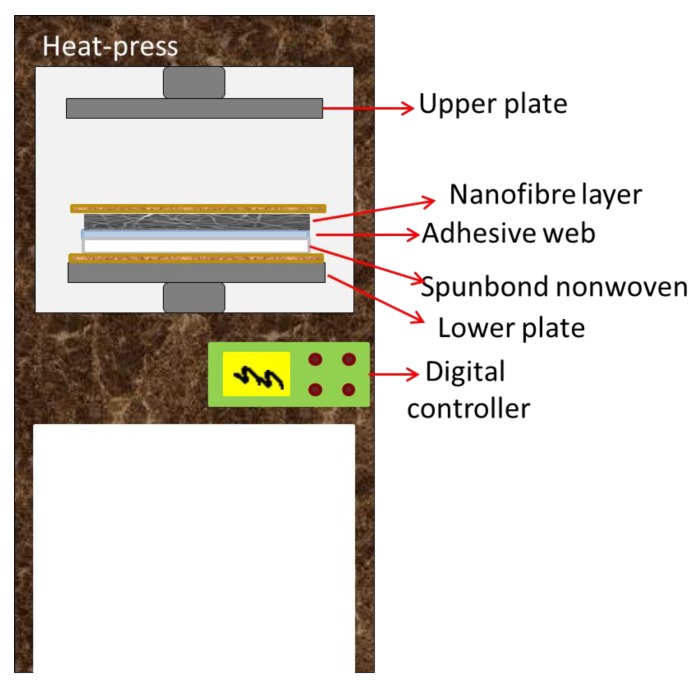
Schematic design of the heat-press equipment and replacement of the multilayer nanofibrous membranes.

The prepared nanofiber webs were cut into A4 size (210 × 297 mm^2^). As a supporting layer, 120 g/m^2^ of polyethylene terephthalate spunbond nonwoven and 12 g/m^2^ of adhesive web were used (the supplier information is not given). Heat-press equipment (Pracovni Stroje, Teplice, Czech Republic) was used for the lamination process (Figure 2). In this equipment there are two metallic hot plates (upper and lower) used under pressure. The nanofibers, a copolyamide adhesive web and a polyethylene terephthalate spunbond supporting layer were placed between the two hot plates. Two silicon layers were used to block direct contact between the multilayer nanofibrous membranes and the hot plates. The heat was applied (130 °C) for a duration of 3 min. Pressures of 50, 75 and 100 kN were applied between the upper and lower plates. For each pressure, PVDF and PAN nanofiber webs were used. The resultant multilayer nanofibrous membranes were termed PAN50, PAN75, PAN100, PVDF50, PVDF75, and PVDF100 according to the pressure value.

### 2.3. Characterization of the Multilayer Nanofibrous Membranes

The surface morphology of the electrospun fibers and laminated multilayer nanofibrous membranes was observed using a scanning electron microscope (SEM, Vega 3SB, Brno, Czech Republic). From each sample, at least 50 fibers were measured. The fiber diameter was analyzed using the Image-J program (free online program). The Origin-Lab program was used to evaluate the diameter distribution. The surface contact angle of the samples was measured using a Krüss Drop Shape Analyzer DS4 (Krüss GmbH, Hamburg, Germany), at five different points, using distilled water (surface tension 72.0 mN m^−1^) and ethylene glycol (surface tension 47.3 mN m^−1^) on the clean and dry samples at room temperature. The air permeability of the multilayer nanofibrous membranes was tested using an SDL ATLAS Air Permeability Tester (@200 Pa and 20 cm^2^, Rock Hill, SC, USA). At least three measurements were taken for each sample.

**Figure 3:**
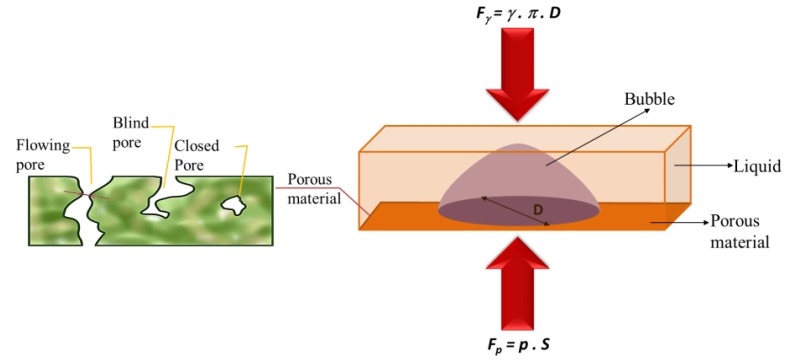
Schematic diagram of pore flow and forces acting on a pore.

The maximum, average and minimum pore sizes were determined by a bubble point measurement device, which was developed in our laboratory. The bubble point test allowed the size of the pores of the porous material to be measured. The pore flow means a set of continuous hole channels connecting the opposite sides of the porous material (see Figure 3). At least three measurements were taken.

The main part of the method was to control the pressure needed to pass a liquid through the tested porous material and for wetting the sample. This is because the wetting force (and hence the opposite force required to extrude the liquid) depends on the pore circumference. The principle for calculating the pore size is shown in Figure 3 and Equations (1) and (2).

*F*_γ_ = γπ*D* (1)

*Fp = pS* (2)

where *F*_γ_ is the force given by surface tension γ of the liquid around the perimeter of π*D*. The force *Fp* is given by external pressure *p* displacing the liquid from the pores and acting on the surface of pore *S*.

**Figure 4:**
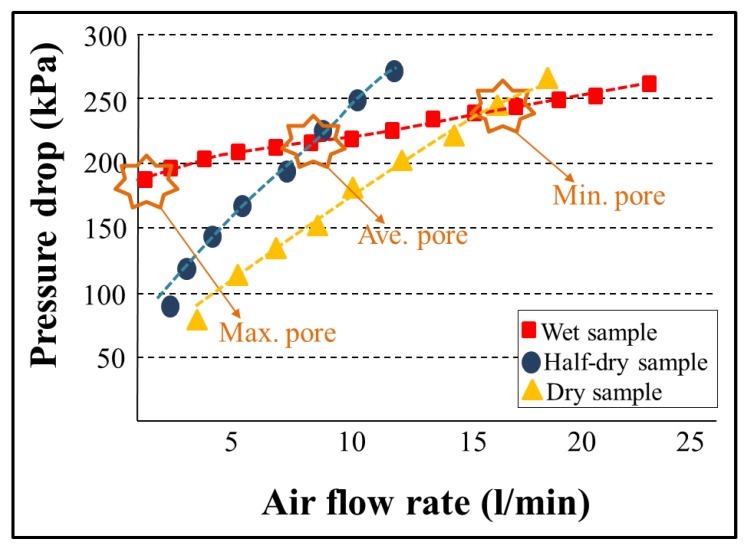
Example of a pressure drop determination to calculate the pore size.

It is possible to calculate the magnitude of the force given by the surface tension and the force given by the pore pressurizing fluid. By increasing the air pressure and measuring its flow through the sample, the size of the average and minimum pores can also be determined. In this case, it is necessary to compare the pressure curve of the wet sample with the pressure curve of the dry sample (see Figure 4). The dry sample pressure curve required to determine the mean and minimum pores is also applicable for determining the air permeability coefficient (*K*) of the sample calculated according to the relation (Equation (3)):

*K* = *Q*/(Δ*p A*) (3)

where *Q* is the air flow rate (m^2^/s), Δ*p* is the pressure drop of the sample, and *A* is the area of air flow (m^2^).

When the pressure increases in the dry sample, the flow rate also increases. Conversely, in the wet sample, at the beginning, there is no flow because all the pores are filled with the liquid. At a certain pressure, the gas empties the largest pore, which determines the minimum pore size, and gas begins to flow through the wet sample. The intersection between the calculated half-dry and the wet sample gives the mean flow pore size. When all the pores are emptied, an intersection between the wet and dry curve will be observed. This means the relation between the applied pressure and the detected flow becomes linear and the intersection of the wet and dry curve represents the detected minimum pore size.

**Figure 5:**
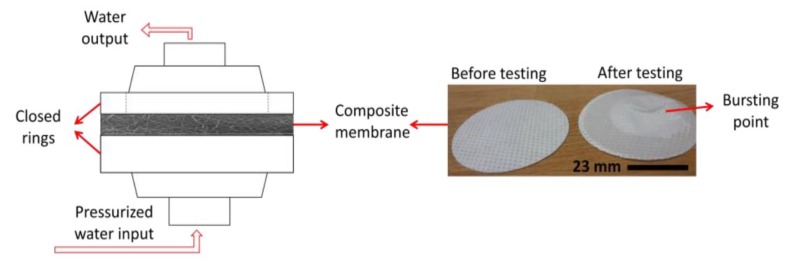
Bursting strength testing unit.

The bursting strength of the multilayer nanofibrous membrane was tested, and the maximum delamination pressure was recorded. The testing device was developed in our laboratory as shown in Figure 5. In this test, the samples were placed between two rings, and the nanofiber side of the samples was placed on the upper side. The sample size was 47 mm in diameter. Pressurized water was sent to the membrane, and the hydrostatic pressure was measured using a pressure controller, which was placed in front of the membrane and connected to a computer. The hydrostatic pressure was increased gradually, and as soon as the nanofiber layer burst, the pressure value on the screen decreased sharply. The maximum pressure value was recorded as the bursting strength of the membrane. The testing samples are shown in Figure 5. After bursting, the nanofiber layer delaminated from the surface of the multilayer membrane. At least three measurements were taken for each membrane.

**Figure 6:**
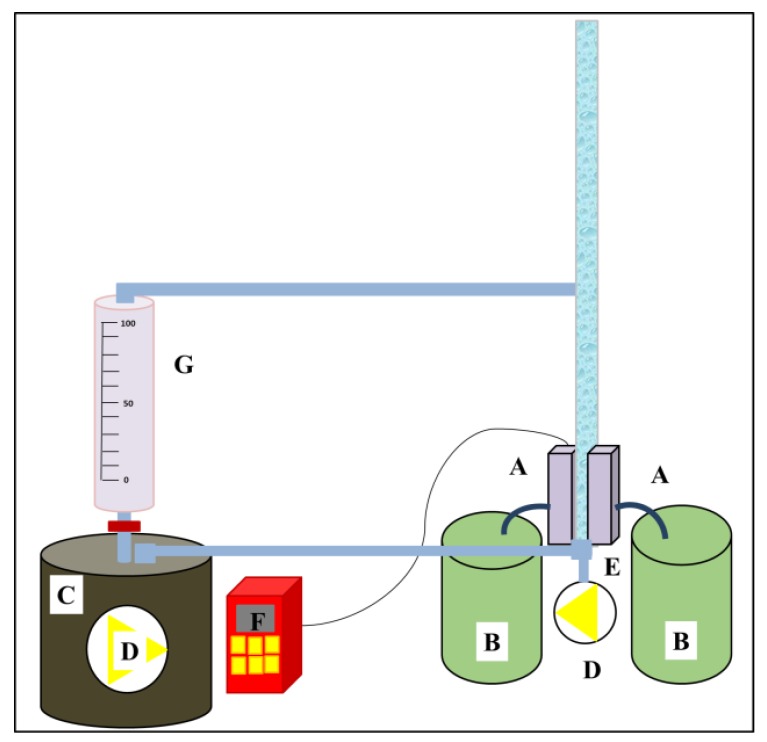
A cross-flow unit: (A) membrane cells; (B) permeate; (C) feed; (D) pump; (E) surface bubble cleaning; (F) pressure controller; (G) feed flow speed controller.

A lab-scale cross-flow filtration unit was developed as shown in Figure 6. Tap water was used as the feed solution. The maximum amount of feed solution was 1500 mL. The flux (*F*) and the permeability (*k*) of the membranes were calculated according to Equations (4) and (5) [31,35]:

(4) $$F=\frac{1}{A}\frac{dV}{dt}$$

(5) $$k=\frac{F}{p}$$

where *A* is the effective membrane area (m^2^), *V* is the total volume of the permeate (*L*), *p* is the transmembrane pressure (bar), and *t* is the filtration time.

## 3. Results and Discussion

### 3.1. Characterization of Nanofibre Webs and Laminated Multilayer Nanofibrous Membranes

To characterize the nanofiber webs into the format of multilayer nanofibrous membranes, various aspects of their material properties were carefully considered. These properties include the fiber diameter, diameter distribution, mean pore size, wetting property, air permeability, and bursting strength.

**Figure 7:**
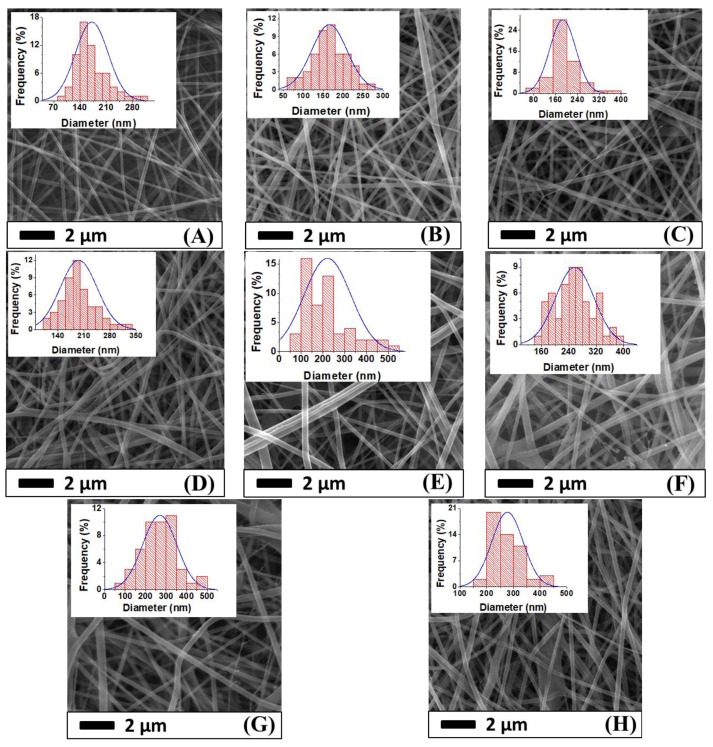
SEM images and fiber diameter distribution of (**A**) PAN nanofiber web before lamination; (**B**) PAN50; (**C**) PAN75; (**D**) PAN100; (**E**) PVDF nanofiber web before lamination; (**F**) PVDF50; (**G**) PVDF75; and (**H**) PVDF100.

**Figure 8:**
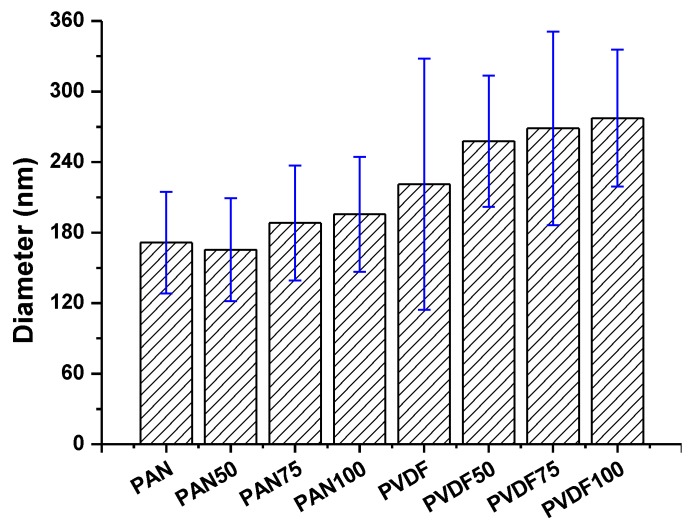
Fiber diameter of various multilayer nanofibrous membranes under different laminating pressures.

The surface morphology of the nanofiber webs before and after lamination was imaged using a scanning electron microscope as shown in Figure 7. The average fiber diameter of the PAN and PVDF nanofibers before lamination was determined to be 171 nm and 221 nm, respectively. The diameter of the PVDF nanofibers was greater than that of the PAN nanofibers. The main reason was the difference in viscosity. In previous work [36], it was determined that a 14 wt. %. PVDF solution has a viscosity of 969 mPa.s, while 8 wt. %. PAN has 191 mPa.s. Based on the viscosity results, one can expect that a polymeric solution with a lower viscosity will have a lower fiber diameter. After the lamination process, neither the PVDF nor the PAN nanofiber diameters significantly changed at a pressure of 50 kN (Figure 8). However, significant changes were observed at laminating pressures of 75 kN and 100 kN. When the highest laminating pressure was applied (i.e., 100 kN), the diameter of the PAN and PVDF nanofibers increased by 14% and 25%, respectively. The fibers were flattened under heat and pressure, and the fiber diameter increased gradually. The highest fiber diameter changes were observed in the case of the PVDF nanofiber layer due to its lower glass transition and melting temperature compared to PAN [37,38]. 

It was verified that there is a strong correlation between the electrospun fiber diameters and the polymer concentration, which has been well documented in the literature [39,40,41]. From the SEM images, the PAN and PVDF multilayer nanofibrous membranes exhibited bead-free surface morphology.

**Figure 9:**
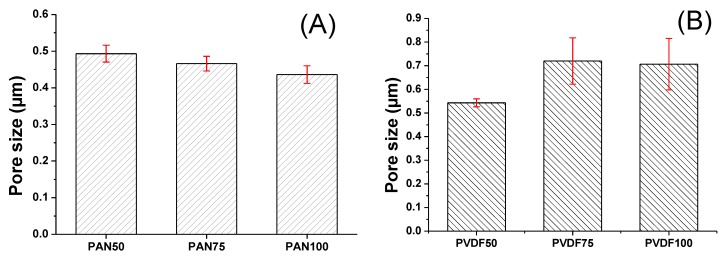
The relationship between the mean pore size and the laminating pressure of (**A**) PAN and (**B**) PVDF multilayer nanofibrous membranes.

The average pore size of the membranes is given in Figure 9. Electrospun materials readily deform at low pressures. Since the tensile strength of PVDF and PAN nanofibers before lamination is quite low to withstand air pressure, their pore size was not measured.

**Figure 10:**
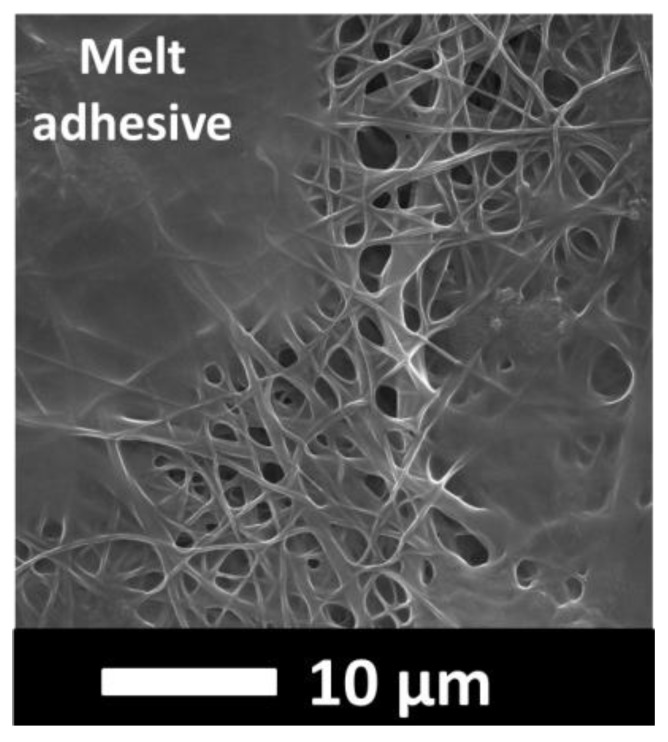
An illustration of adhesive melting over the surface of a nanofiber web, forming a non-porous film.

In general, there is a correlation between the fiber diameter and the average pore size of the nanofibers. Reducing the fiber diameter increases the surface area and compact web structure, which results in a small pore size [42]. Bagherzadeh et al. [43] demonstrated a theoretical analysis to predict the pore size of electrospun nanofibers. According to their theory, at a given web porosity, increasing the fiber diameter and thickness of the web reduces the dimensions of the pores. This theory was validated experimentally, and the results were compared with the existing theory to predict the pore size distribution of nanofiber mats. Their results showed that the pore size significantly increased with an increase in fiber diameter, web porosity and density of the layers. In this work, the correlation between the diameter of the PVDF nanofibers and the mean pore size was compatible with the literature, while PAN showed an opposite correlation. The laminating pressure effect must be taken into consideration. The nanofiber layer did not change; only the fibers flattened after lamination due to the pressure. It was expected that a higher pressure would cause a lower pore size since the fibers flattened and melting adhesive filled more of the pores and covered the surface of the nanofibers as shown in Figure 10. The PAN multilayer nanofibrous membranes fulfilled this expectation while the PVDF did not. Figure 9B shows that the average pore size and the standard deviation of the pore size measurements increased with pressure, which could be due to possible damage of the PVDF nanofibers under high pressure. Gockeln et al. [44] investigated the influence of laminating pressures on the microstructure and electrochemical performance of the lithium-ion battery electrodes. The results indicated that all the laminated samples showed highly porous and homogeneous networks, while the pore size slightly decreased with an increase in laminating pressure. At higher pressures, the intrinsic electrical conductivity was improved due to more compression.

**Figure 11:**
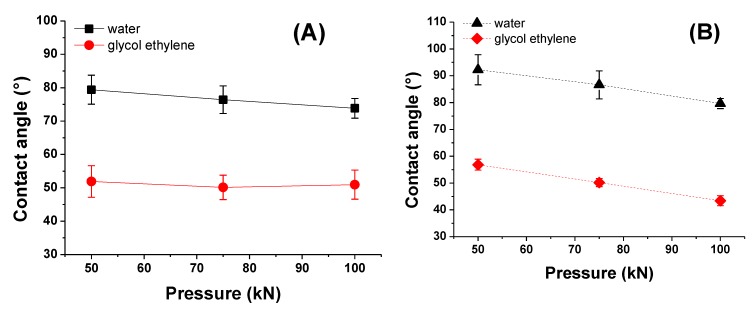
Contact angle vs. laminating pressure of (**A**) PAN; (**B**) PVDF multilayer nanofibrous membranes.

The water and ethylene glycol wettability of the PAN and PVDF multilayer nanofibrous membranes were examined by a contact angle measurement as shown in Figure 11. The surface energy and surface roughness are the dominant factors for the wettability. As can be seen from Figure 11, an increase in laminating pressure decreased the water and ethylene glycol contact angle of both PAN and PVDF multilayer nanofibrous membranes. Hence, ethylene glycol has a lower surface energy compared to water, with the differences in contact angle value being 20° for PAN and 30° for PVDF. Similar behavior was observed in the literature [45,46]. It is well known that when the surface energy is lowered, and surface roughness is raised, the hydrophobicity is enhanced [47,48,49]. With the help of heat, the higher laminating pressure on the surface may cause changes to the surface shape and make the surface flatter, which results in an increase in the surface wettability (Figure 11). The PVDF membranes showed hydrophobic characteristics at the lowest laminating pressure (i.e., 50 kN), while at higher laminating pressures they showed hydrophilic properties. By setting the lamination process parameters, one can prepare hydrophilic PVDF multilayer nanofibrous membranes without any surface modification.

**Figure 12:**
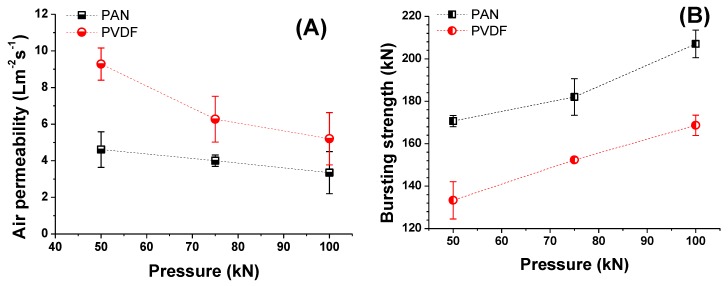
Influence of laminating pressure on (**A**) air permeability; and (**B**) bursting strength of multilayer nanofibrous membranes.

The morphology of the nanofiber webs, including their pore size, shape, size distribution and porosity, has a significant influence on the air permeability of the multilayer membrane. To investigate the effect of laminating pressure on the air permeability of the multilayer nanofibrous membranes, the samples were placed on a circular sample holder, and the air flow rates through the samples were measured (Figure 12A). Like the air flows, the areas of the sample and pressure drop remained constant during the measurement. Due to the weakness of neat nanofiber layers, the air permeability test was not performed. In a previous study [31], the tensile strength of the nanofiber layers was found to be between 3 and 4.33 (N/25 mm), which is extremely low to withstand any external force.

Abuzade et al. [50] studied the effects of the process parameters (e.g., concentration of solution, applied voltage) on the porosity and air permeability of an electrospun nanoweb. The results showed that the nanofiber diameter and size distribution are dominant parameters in controlling the pore sizes formed by the nanofiber intersections and air permeability of the electrospun web. Figure 12A showed that increasing the laminating pressure lowered the air permeability of the multilayer membranes. Compression of the melting adhesive, filling the pores of the nanofiber and nonwoven web, covered the surface of the thin nanofiber layer and created a non-porous film (Figure 10). As a result, the breathability of the membranes was decreased. Similarly, Kanafchian et al. [32] claimed that during the lamination, the melt adhesive penetrates through the nanofiber/fabric structure, which leads to filling of the pores of nanofibers and a decrease in air permeability. The PAN multilayer nanofibrous membrane has a lower air permeability than PVDF, mainly due to the lower fiber diameter of the PAN nanofibers compared to the PVDF nanofibers. Rajak et al. [51] prepared PAN nanofiber webs from various concentrations. The results indicated that changes in concentration affect the fiber diameter. At a higher concentration and fiber diameter, the air permeability has a higher value.

A bursting test was performed to determine the mechanical strength of the laminated layers, and the results are shown in Figure 12B. The test method has been developed in our laboratory. The maximum delamination point of the multilayer nanofibrous membranes was measured using hydrostatic pressure. The results showed that PAN nanofibers have a better adhesion to the supporting layer and a better bursting strength compared to PVDF. The adhesion between the layers is related to the material surface chemistry and its influence on adhesion, together with the properties of adhesive materials and interactions at the adhesive-substrate surface interface. Materials that can wet each other tend to have a better adhesion, and the wettability of the material is related to its surface energy. For instance, low surface energy materials such as poly(tetrafluoroethylene), ceramics, and silicon, are resistant to wetting and adhesive bonding [52]. Lee et al. [53] found that the surface energy of PAN is around 44 mJ/m^2^, while this value was calculated as 54.1 mJ/m^2^ by Pritykin et al. [54]. On the other hand, PVDF has a very low surface energy value of around 26 mJ/m^2^ [55]. Due to the lower surface energy of PVDF compared to PAN, the adhesion between the layers is weaker, which results in low lamination strength. The results show that laminating pressure plays an important role in the bursting strength. By increasing the laminating pressure under heat, the melted adhesive fills the pores of the nanofibers and nonwovens and penetrates through the layers. A better mechanical strength is achieved due to the entanglement of the adhesive web and the layers. The results showed that the bursting strength of a material can be improved by adjusting the lamination conditions. 

### 3.2. Evaluation of Liquid Filtration by Cross-Flow Filtration

**Figure 13:**
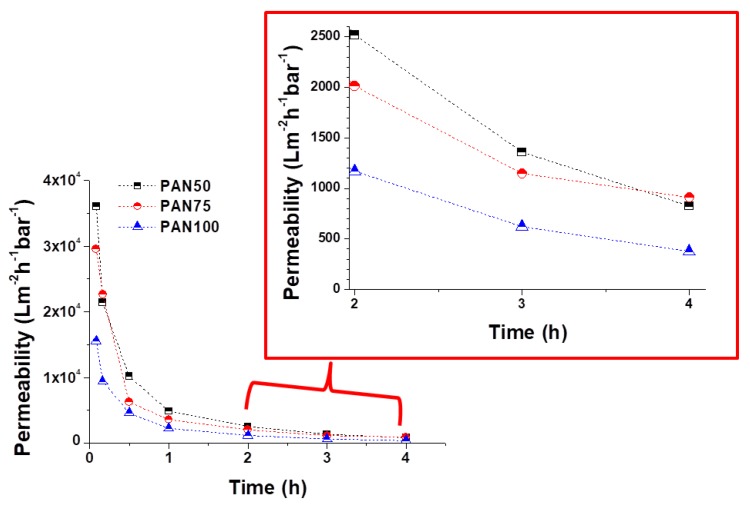
Permeability of PAN multilayer nanofibrous membranes at various laminating pressures over time.

**Figure 14:**
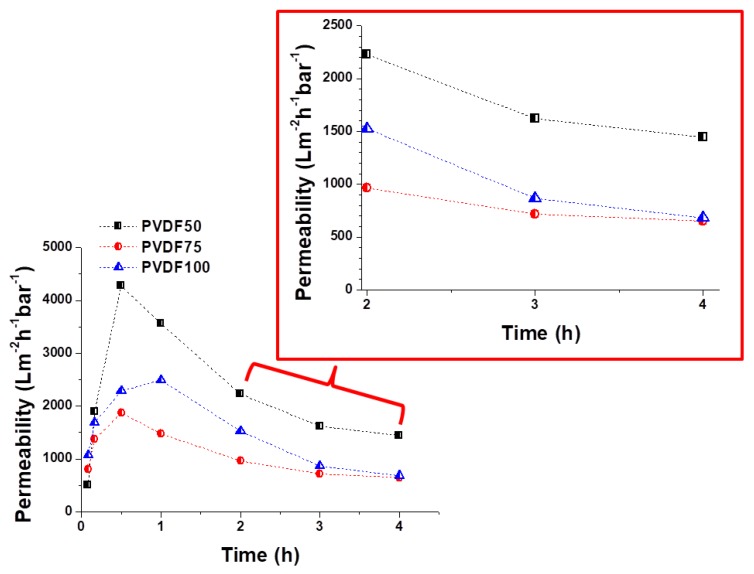
Permeability of PVDF multilayer nanofibrous membranes at various laminating pressures over time.

Taking their practical applications into consideration, laminated multilayer nanofibrous membranes were used to further investigate their water permeability performance due to their hydrophilic, porous, small pore size and predominant mechanical properties for liquid filtration. A cross-flow filtration unit was prepared in our laboratory. Using Equation (5), the water permeability of the PAN and PVDF multilayer nanofibrous membranes was calculated (Figure 13 and Figure 14).

A decrease in permeability was observed for both the PAN and PVDF multilayer nanofibrous membranes depending on the operation time as shown in Figure 13 and Figure 14. There are a few possible reasons for the decrease in permeability during liquid filtration. The first reason is concentration polarization, which is a consequence of the selectivity of the membrane. When the liquid passes through the membrane, the solute is retained by the membrane surface with a relatively high concentration. Moreover, the hydrophilicity of the membrane decreases over time during filtration due to membrane fouling and concentration polarization. Since tap water is not pure, dissolved molecules, suspended solids, and organics may be contained in the water, which can cause a decrease in the water flux due to fouling. The second reason is that close to the membrane surface, the effective transmembrane pressure (TMP) driving force reduces due to an osmotic pressure difference between the filtrate and the feed solution. TPM is generally observed in the case of ultra-filtration (UF) membranes. Another reason may be related to the compression/collapse of membrane pores, thereby causing a reduction in water permeability. The operating conditions (feed pressure, temperature, pH, flow rate, etc.) are also effective factors in membrane permeability. In general, the flux decline is caused by a decreased driving force and/or an increased resistance of the membrane, raw water characteristics, and particulate matter levels [56,57,58].

At the beginning, all the PAN membranes had the highest permeability (Figure 13). After a 4-h filtration test, the flux declined to 824, 909, and 375 Lm^−2^h^−1^bar^−1^ in the case of PAN50, PAN75, and PAN100, respectively. The results indicated that laminating pressure has a huge impact on the water permeability of the multilayer membranes. The laminating pressure and the permeability of the membranes showed a non-linear relationship in the case of the PAN membranes. PAN50 and PAN75 multilayer nanofibrous membranes showed the best water permeability. On the other hand, all the PVDF membranes showed very low initial permeability at the beginning due to the hydrophobic nature of the PVDF nanofibers, and the melted adhesive web partially occupied the membrane pores, increasing the hydraulic resistance to filtration (Figure 14). The results of 4 h of filtration of PVDF membranes showed that the highest permeability (1444 Lm^−2^h^−1^bar^−1^) was only achieved at the lowest laminating pressure (50 kN). PVDF75 and PVDF100 had almost the same permeability value (650 and 681 Lm^−2^h^−1^bar^−1^, respectively) after the 4-h filtration test. Li et al. [59] reported a simple strategy to improve the waterproof/breathable performance and mechanical properties of electrospun PVDF fibrous membranes using a thermo-pressing system. It was found that the effect of temperature and pressure on PVDF has a synergistic effect on the fiber morphology and crystal structure. By properly adjusting the temperature and pressure, robust mechanical properties and excellent waterproof/breathable performance of PVDF membranes were achieved. 

In terms of water permeability, PAN75 has the best results from the PAN membranes. PVDF50 showed the best permeability results after the 4-h filtration test from all the PAN and PVDF membranes. The results showed that after proper lamination multilayer nanofibrous membranes are suitable for future application in liquid filtration.

## 4. Conclusions

There is a huge demand for the filtration application of nanofiber layers due to their specific surface, low pore size and high porosity. In this study, the effect of laminating pressure on PAN and PVDF multilayer nanofibrous membranes was investigated to prepare suitable microfilters for liquid filtration. The surface morphology, average pore size, air permeability, water permeability, bursting strength, and the contact angle of the membranes were compared. Different performance levels were achieved by varying the laminating pressure of the multilayer nanofibrous membranes. The pressure effect had a considerable influence on air permeability, average pore size, contact angle, bursting strength, and water permeability. The surface morphology results showed that the fiber diameter slightly increased with an increase in laminating pressure, while the water and ethylene glycol contact angles decreased. The main effect of laminating pressure was observed on the average pore size, air permeability, bursting strength and water permeability of the membranes. PVDF50 showed the best water filtration of all the membranes. However, the bursting strength of PVDF50 is the lowest, which may cause possible damage and delamination of the layers under pressure over time. PAN nanofibers have a better adhesion to the surface of the multilayer. PAN75 was selected as the best candidate for liquid filtration due to its high water permeability and mechanical strength. PVDF multilayer nanofibrous membranes showed better air permeability than PAN, which may be better for the possible application of air filtration. These findings imply that to achieve the best permeable membrane results, the lamination process should be carefully optimized.
